# Depolarization time and extracellular glutamate levels aggravate ultraearly brain injury after subarachnoid hemorrhage

**DOI:** 10.1038/s41598-022-14360-1

**Published:** 2022-06-17

**Authors:** Satoshi Murai, Tomohito Hishikawa, Yoshimasa Takeda, Yasuko Okura, Miki Fushimi, Hirokazu Kawase, Yu Takahashi, Naoya Kidani, Jun Haruma, Masafumi Hiramatsu, Kenji Sugiu, Hiroshi Morimatsu, Isao Date

**Affiliations:** 1 Department of Neurological Surgery, Okayama University Graduate School of Medicine, Dentistry and Pharmaceutical Sciences, Okayama, Japan; 2 Department of Anesthesiology, Omori Medical Center, Toho University, Tokyo, Japan; 3 Department of Anesthesiology and Resuscitology, Okayama University Graduate School of Medicine, Dentistry and Pharmaceutical Sciences, Okayama, Japan

**Keywords:** Stroke, Experimental models of disease

## Abstract

Early brain injury after aneurysmal subarachnoid hemorrhage (SAH) worsens the neurological outcome. We hypothesize that a longer duration of depolarization and excessive release of glutamate aggravate neurological outcomes after SAH, and that brain hypothermia can accelerate repolarization and inhibit the excessive release of extracellular glutamate and subsequent neuronal damage. So, we investigated the influence of depolarization time and extracellular glutamate levels on the neurological outcome in the ultra-early phase of SAH using a rat injection model as Experiment 1 and then evaluated the efficacy of brain hypothermia targeting ultra-early brain injury as Experiment 2. Dynamic changes in membrane potentials, intracranial pressure, cerebral perfusion pressure, cerebral blood flow, and extracellular glutamate levels were observed within 30 min after SAH. A prolonged duration of depolarization correlated with peak extracellular glutamate levels, and these two factors worsened the neuronal injury. Under brain hypothermia using pharyngeal cooling after SAH, cerebral perfusion pressure in the hypothermia group recovered earlier than that in the normothermia group. Extracellular glutamate levels in the hypothermia group were significantly lower than those in the normothermia group. The early induction of brain hypothermia could facilitate faster recovery of cerebral perfusion pressure, repolarization, and the inhibition of excessive glutamate release, which would prevent ultra-early brain injury following SAH.

## Introduction

Aneurysmal subarachnoid hemorrhage (SAH) remains a devastating and challenging disease with high morbidity and mortality^1^. Approximately 30% of patients develop delayed cerebral ischemia (DCI)^2^, resulting in unfavorable neurological outcomes^3^. In recent years, early brain injury (EBI) has been considered a major etiology of DCI. EBI involves pathophysiological changes triggered by the abrupt increase of intracranial pressure and subsequent transient global ischemia, and it occurs within 72 h after SAH^4^. Various mechanisms are involved in EBI, including cortical spreading depolarization (CSD)^5^, neuronal inflammation^6^, microcirculation impairment, blood–brain barrier disruption^7^, and hypercoagulability^8^.

It is known that in the acute-phase of SAH, intracranial pressure (ICP) and cerebral blood flow (CBF) changes dynamically, resulting in excessive release of excitatory amino acids such as glutamate, which causes neuronal damage^9^. Shimizu et al.^10^ divided the changes in extracellular potentials into three types, and showed that the duration of depolarization was significantly correlated with the severity of neurological injury in a rat perforation model. They also revealed that the duration of depolarization that caused 50% of neuronal damage was estimated to be 22.4 min, and hypothesized that controlling the ICP within the first hour after SAH was important for brain protection. Although both the duration of depolarization and extracellular glutamate levels are associated with hyperacute neuronal damage, the detailed mechanisms of how these factors interact and the recovery process of membrane potentials and the changes in other physiological parameters after SAH are not well known.

We have focused on a neuroprotective effect of brain hypothermia for EBI. Brain hypothermia has a neuroprotective effect through reducing oxygen demand, decreasing blood–brain barrier permeability, limiting inflammatory cell entry, and decreasing calcium-dependent cell injury^11^. We hypothesize that brain hypothermia contributes to lowering energy consumption, accelerating repolarization, which inhibits the excessive release of extracellular glutamate and subsequent neuronal damage. But there are no reports investigating the duration of depolarization and extracellular glutamate level simultaneously after induction of brain hypothermia.

To investigate the effect of the duration of depolarization and the extracellular glutamate levels on EBI, and a neuroprotective effect of the brain hypothermia, we conducted two experiments. In Experiment 1, we evaluated the changes in physiological parameters in the ultra-early phase of SAH, including loss of membrane potentials, mean arterial pressure (MAP), ICP, cerebral perfusion pressure (CPP), and CBF. We also closely investigated the relationship between the duration of depolarization and the extracellular glutamate levels. Next, the neurological and histological outcomes were evaluated. In Experiment 2, we investigated the effect of brain hypothermia on neurological and histological outcomes in terms of the changes in the physiological parameters and glutamate release.

## Results

### Experiment 1

#### Characteristics

The baseline results of ABG are shown in Supplementary Table 1. The CO_2_ level was well controlled (37.5 ± 3.3 mmHg). The mean volume of injection blood was 0.34 ± 0.03 ml. Cortical depolarization was observed in 8 (88.9%) rats, and mean depolarization was 20.6 min. A depolarization duration of more than 20 min was observed in 4 (44.4%) rats (Supplementary Table 2). One of the nine rats (11%) died within 24 h after SAH.

#### Temporal changes of physiological parameters

**Figure 1:**
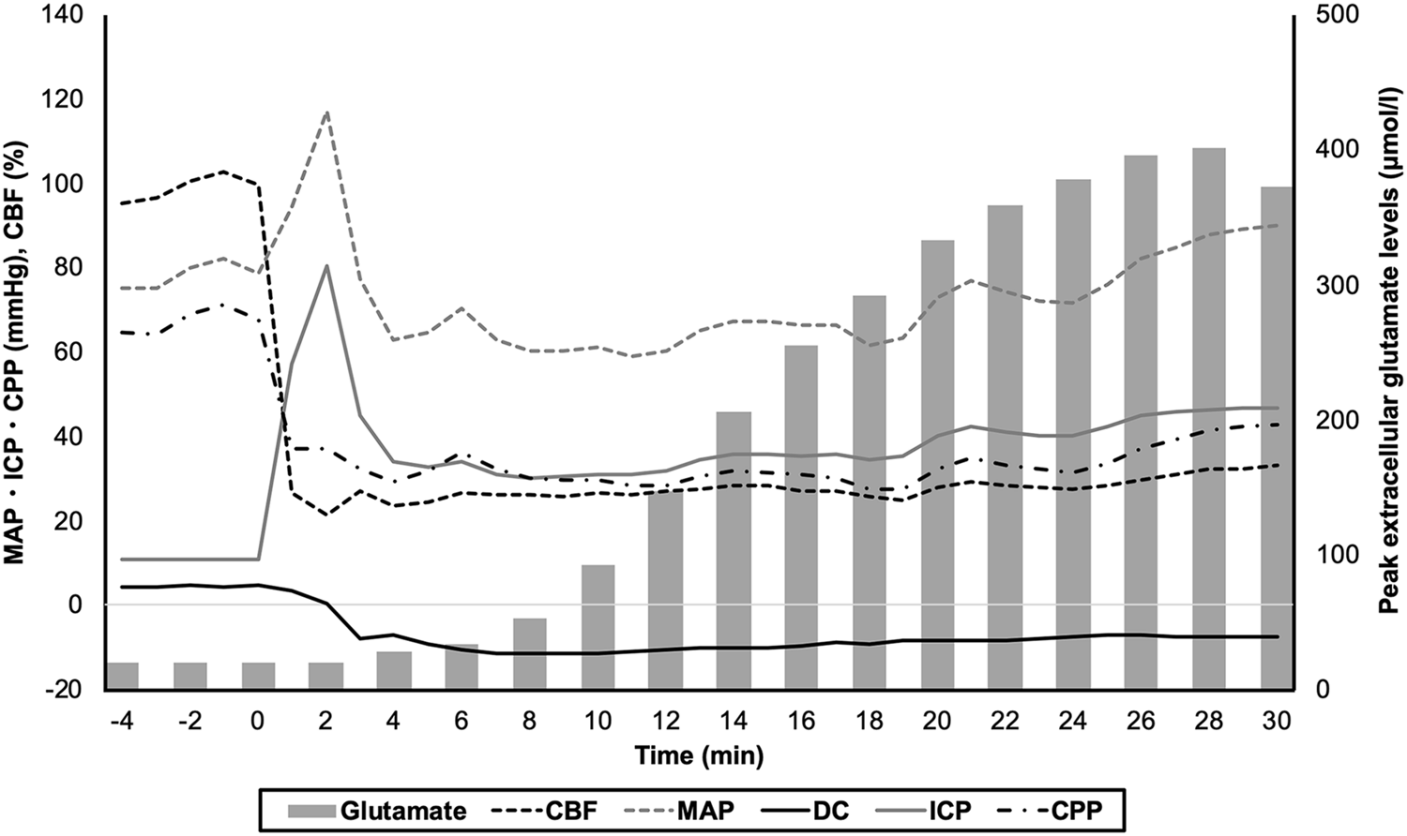
The changes in the physiological parameters in the illustrative case with a longer duration of depolarization. Cortical depolarization occurred within 2 min after blood injection. ICP abruptly elevated immediately after injection. It transiently decreased to around 30 mmHg within 10 min, and gradually increased during the observation period. MAP increased immediately after injection along with ICP elevation, and recovered to the normal level within 4 min. CPP declined immediately after injection along with ICP elevation, and was under 40 mmHg at 1 min. CBF also decreased immediately after injection, and reached 30% of the baseline. CPP and CBF gradually recovered during 30 min. The extracellular glutamate levels started to rise from 10 min after blood injection, and plateaued at 20 min.

Dynamic changes in membrane potentials, MAP, ICP, CPP, CBF, and extracellular glutamate levels were observed during the first 30-min observation. Cortical depolarization occurred within 2 min after blood injection. ICP abruptly elevated immediately after injection. It transiently decreased to around 30 mmHg within 10 min, and showed a gradual increase during the observation period. MAP increased immediately after injection along with ICP elevation. It recovered to the normal level within 4 min. CPP declined immediately after injection along with ICP elevation, and was under 40 mmHg at 1 min. It recovered to approximately 50 mmHg within 10 min, but gradually decreased again. CBF also decreased immediately after injection, and reached 25 to 50% of the baseline. It recovered to approximately 40 to 80% of baseline within 10 min, then gradually declined and hovered around 20 to 60%. The extracellular glutamate levels started to rise from 10 min after blood injection, and plateaued at 20 min. The changes in the physiological parameters in the illustrative case with a longer duration of depolarization are shown in Fig. 1.

**Figure 2:**
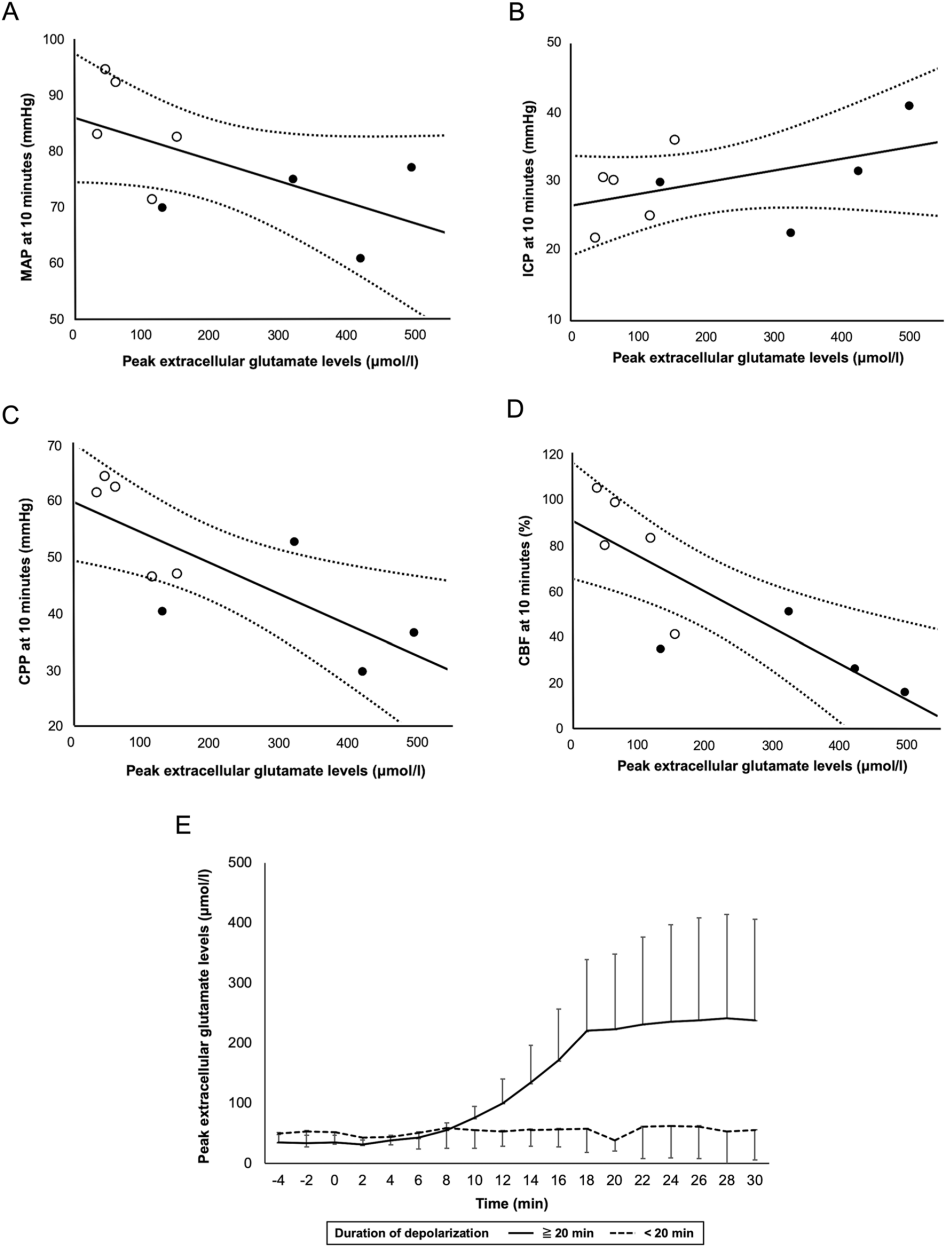
Linear regression analysis showed that MAP and ICP at 10 min were not correlated with peak extracellular glutamate levels (*r* = -0.59, *p* = 0.089 and *r* = 0.47, *p* = 0.20, respectively) (**A**, **B**), but CPP at 10 min and CBF at 10 min were significantly correlated with peak extracellular glutamate levels (*r* = -0.77, *p* = 0.016 and *r* = -0.82, *p* = 0.0073, respectively) (**C**, **D**) (Black circles mean the duration of depolarization 20 min or more, and open circles mean the duration of depolarization less than 20 min). When the duration of depolarization was 20 min or more, the extracellular glutamate levels started to rise 10 min after blood injection, and almost plateaued at 20 min (**E**).

Linear regression analysis showed that MAP and ICP at 10 min were not correlated with peak extracellular glutamate level (*r* = −0.59, *p* = 0.089 and *r* = 0.47, *p* = 0.20, respectively), but CPP and CBF at 10 min were significantly correlated with peak extracellular glutamate levels (*r* = −0.77, *p* = 0.016, and *r* = −0.82, *p* = 0.0073, respectively) (Fig. 2A–D). At 20 min after SAH, MAP, ICP, and CPP were not correlated with extracellular glutamate level (*r* = 0.13, *p* = 0.73, *r* = 0.51, *p* = 0.16, and *r* = −0.21, *p* = 0.58, respectively), and only CBF was significantly correlated with peak extracellular glutamate levels (*r* = −0.87, *p* = 0.0022) (Supplementary Fig. 1). Similarly, at 30 min after SAH, MAP, ICP, and CPP were not correlated with extracellular glutamate level (*r* = 0.045, *p* = 0.91, *r* = 0.57, *p* = 0.11, and *r* = −0.45, *p* = 0.23, respectively), and only CBF was significantly correlated with peak extracellular glutamate levels (*r* = −0.87, *p* = 0.0020) (Supplementary Fig. 2).

#### Extracellular glutamate levels and neuronal injury

The extracellular glutamate levels at baseline were 44.0 ± 17.2 μmol/l. From our previous findings, we compared the extracellular glutamate levels between the two groups: duration of depolarization 20 min or more and less than 20 min. When the duration of depolarization was 20 min or more, the extracellular glutamate levels started to rise 10 min after blood injection, and almost plateaued at 20 min. When the duration of depolarization was less than 20 min, the extracellular glutamate levels were almost unchanged during the 1-h observation (Fig. 2E). The peak extracellular glutamate level in the former group was 345.7 ± 159.4 μmol/l, which was significantly higher than 83.6 ± 49.9 μmol/l in the latter group (*p* = 0.0097).

**Figure 3:**
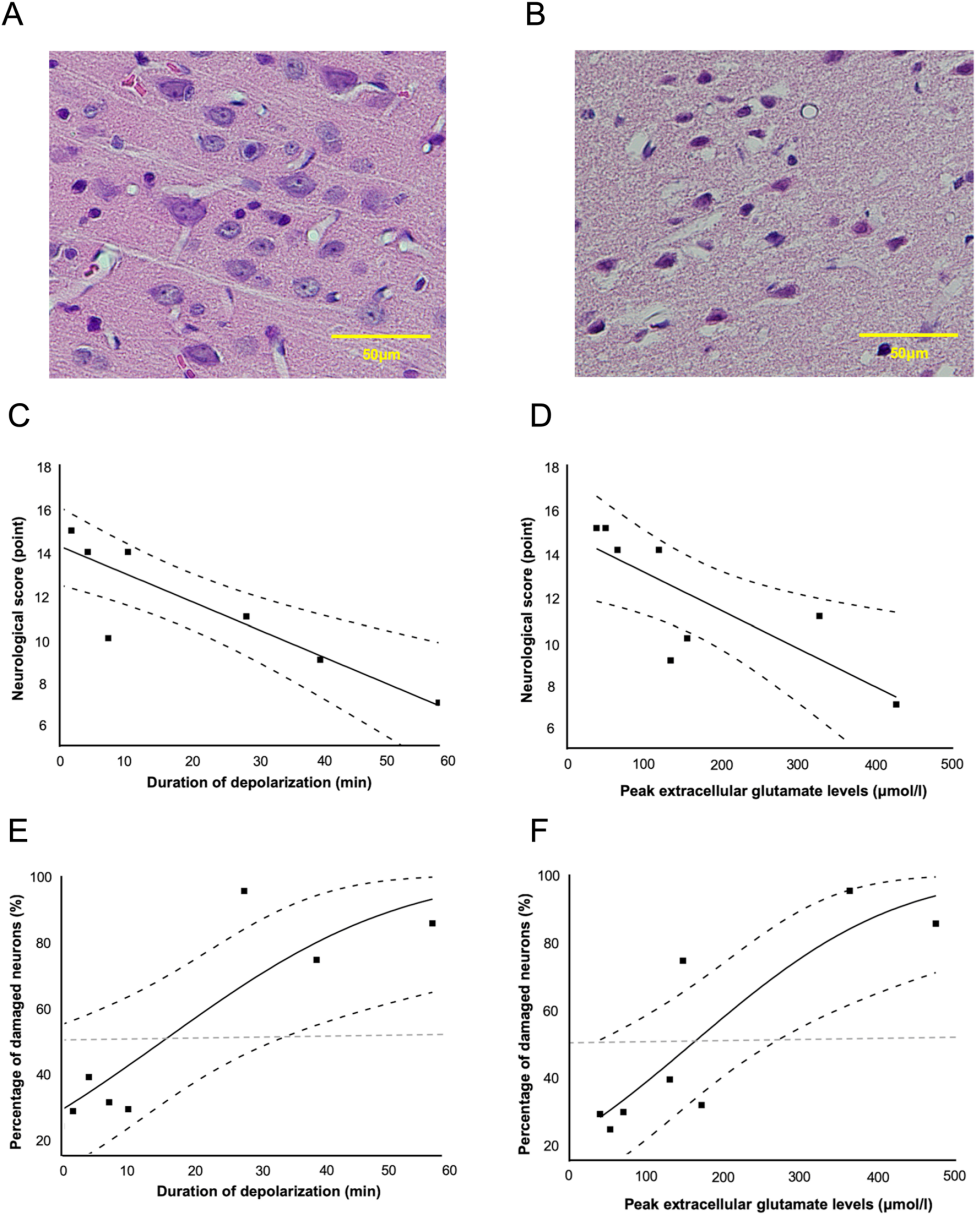
Histologically, cases with longer duration of depolarization showed severe neuronal damage characterized by chromatin aggregation in the nucleus, shrinkage, or eosinophilic staining in the cytoplasm (**A**), and cases with short duration of depolarization showed mild neuronal damage (**B**). Duration of depolarization (**C**) and extracellular glutamate levels (**D**) were significantly correlated with neuronal injuries (*r* = -0.89, *p* = 0.0033, and *r* = -0.80, *p* = 0.018). Probit analysis showed that the duration of depolarization (**E**) and extracellular glutamate levels (**F**) that caused 50% of neuronal damage were 16.5 min and 146 μmol/l, respectively.

Histologically, cases with short duration of depolarization showed mild neuronal damage, but cases with longer duration of depolarization showed severe neuronal damage characterized by chromatin aggregation in the nucleus, shrinkage, or eosinophilic staining in the cytoplasm (Fig. 3A, B). The duration of depolarization was significantly correlated with extracellular glutamate levels (*r* = 0.77, *p* = 0.016). Both the duration of depolarization and extracellular glutamate levels were negatively correlated with neurological score (*r* = −0.89, *p* = 0.0033, and *r* = −0.80, *p* = 0.018) (Fig. 3C, D). Probit analysis showed that the duration of depolarization was significantly correlated with neuronal damage (*r* = 0.81, *p* = 0.014), and the value that caused 50% of neuronal damage was estimated to be 16.5 min (Fig. 3E). The peak extracellular glutamate levels were significantly correlated with neuronal damage (*r* = 0.85, *p* = 0.0073), and the value that caused 50% of neuronal damage was estimated to be 146.1 μmol/l (Fig. 3F).

### Experiment 2

#### Characteristics

**Figure 4:**
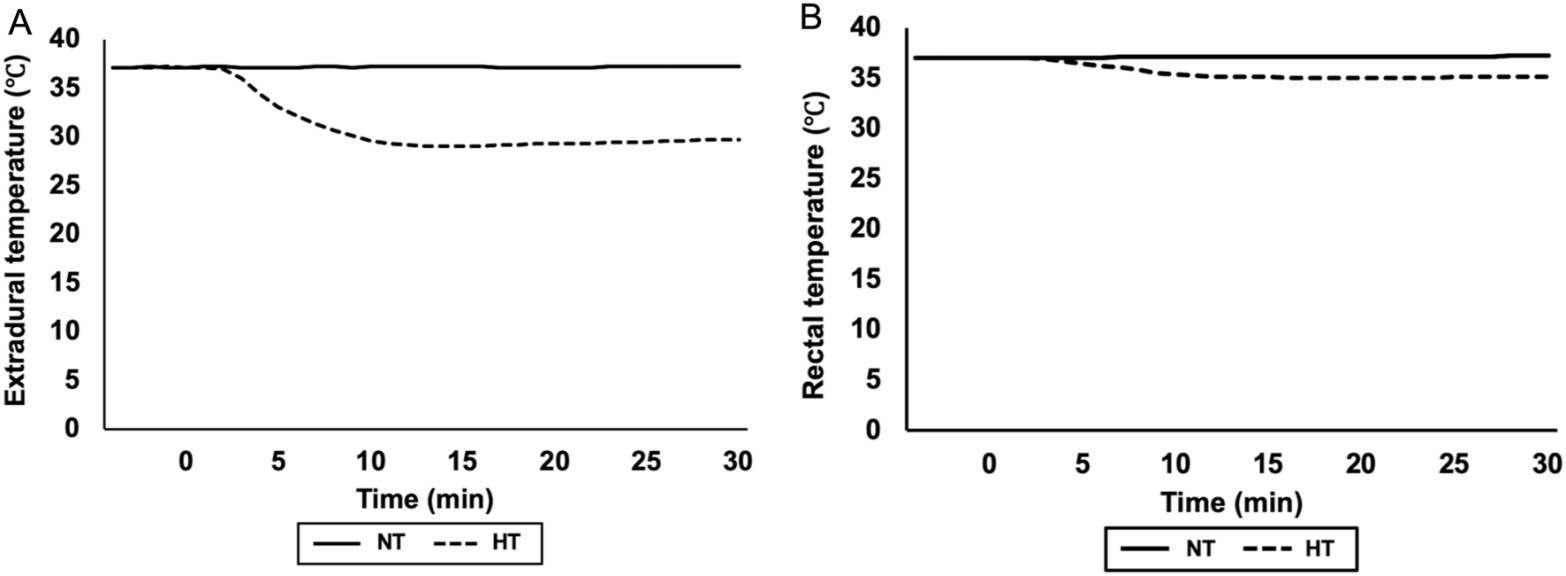
After pharyngeal cooling, the epidural temperature immediately declined to 30 °C within 7 min (**A**). In contrast, the rectal temperature was preserved within 35 °C (**B**).

The baseline results of ABG are shown in Supplementary Table 3. The CO_2_ level was well controlled (39.0 ± 2.7 mmHg). The mean volume of injection blood was 0.36 ± 0.04 ml. Cortical depolarization was observed in 7 (100%) rats, and the mean duration of depolarization was 13.0 ± 17.8 min. A depolarization duration of more than 20 min was observed in 2 (28.6%) rats (Supplementary Table 4). Pharyngeal cooling was started immediately after depolarization. The epidural temperature decreased and reached 30 °C in 7.0 ± 1.6 min. The minimum epidural temperature was 28.9 ± 0.3 °C, and the average final epidural temperature was 30.1 ± 0.7 °C (Fig. 4A). In contrast to the epidural temperature, the rectal temperature was preserved within 35 °C (Fig. 4B). One of the seven rats (14.3%) died within 24 h after SAH.

#### Temporal changes of physiological parameters

**Figure 5:**
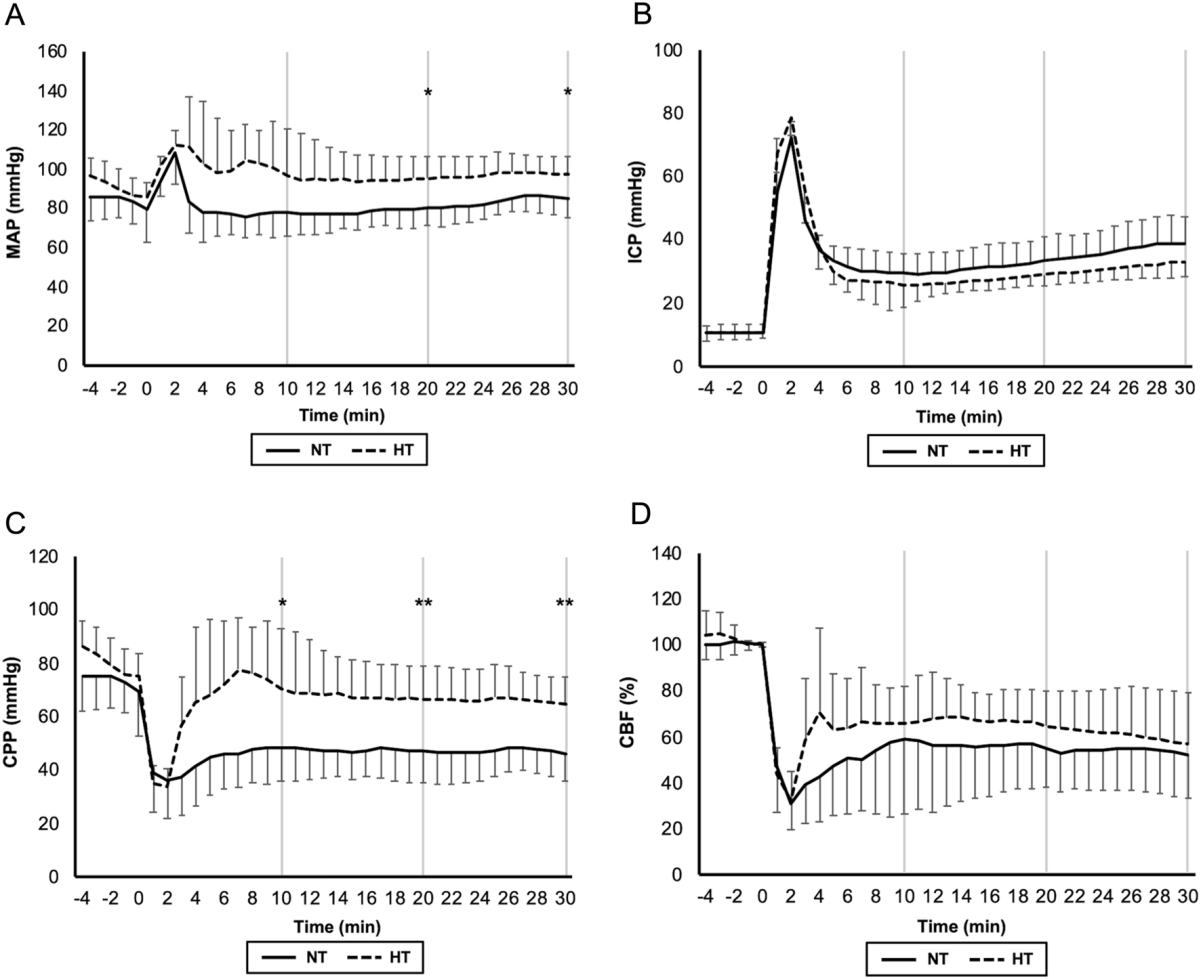
MAP elevated immediately after blood injection in both groups. It decreased to baseline within 4 min in the NT group, but remained higher and hovered around 100 mmHg in the HT group (**A**). ICP abruptly elevated immediately after blood injection. It decreased to under 30 mmHg within 10 min, and gradually increased again in both groups (**B**). CPP declined to 40 mmHg in the NT group and 30 mmHg 1 min after blood injection. CPP in the NT group remained low around 40 mmHg, but that in the HT group recovered to 70 mmHg within 10 min, and gradually declined again. The difference between the two groups was significant at 10, 20, and 30 min (**C**). CBF decreased to 30% from baseline in both groups immediately after blood injection. CBF in the HT group recovered to 60% within 10 min, which was earlier than that in the NT group. Then, it declined again in both groups (**D**) (*p < 0.05, **p < 0.01).

MAP elevated immediately after blood injection in both groups. It decreased to baseline within 4 min in the NT group, but remained higher and hovered around 100 mmHg in the HT group. The difference between the two groups was significant at 20 and 30 min (*p* = 0.015 and *p* = 0.015, respectively) (Fig. 5A). ICP abruptly elevated immediately after blood injection. It decreased to less than 30 mmHg within 10 min, and gradually increased again in both groups. It was slightly lower in the HT group at 10, 20, and 30 min, but the difference between the two groups was not significant (*p* = 0.26, 0.17, and 0.17, respectively) (Fig. 5B). CPP declined to 40 mmHg in the NT group and 30 mmHg in the HT group 1 min after blood injection. CPP in the NT group remained low around 40 mmHg, but that in the HT group recovered to 70 mmHg within 10 min, and gradually declined again. The difference between the two groups was significant at 10, 20, and 30 min (*p* = 0.030, 0.0083, and 0.0034, respectively) (Fig. 5C). CBF decreased to 30% from baseline in both groups immediately after blood injection. CBF in the HT group recovered to 60% within 10 min, which was earlier than that in the NT group. Then, it declined again in both groups. The difference was not significant between the two groups at 10, 20 and 30 min (*P* = 0.63, 0.36, and 0.64, respectively) (Fig. 5D).

#### Extracellular glutamate levels and neuronal injury

**Figure 6:**
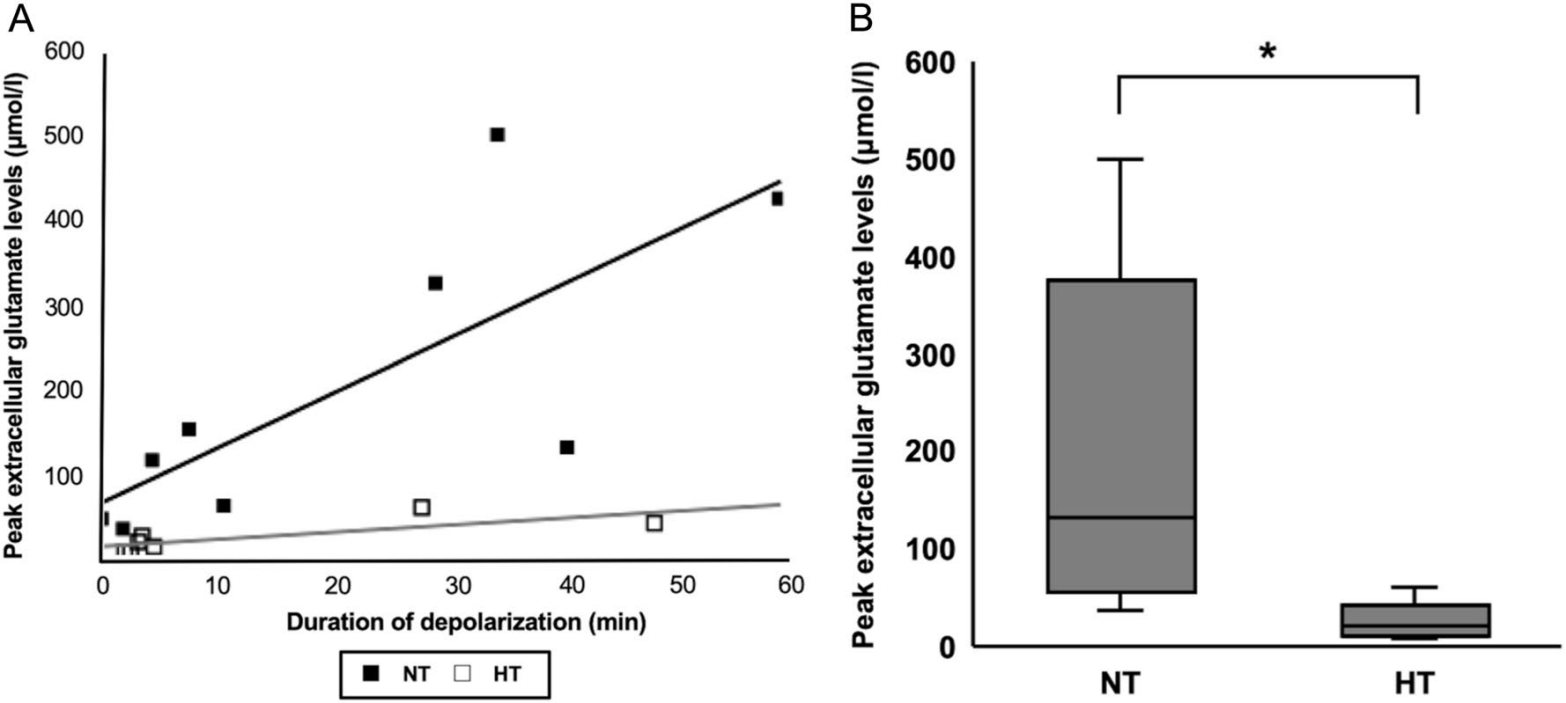
In the NT group, peak extracellular glutamate levels elevated in proportion to the duration of depolarization, but in the HT group, peak extracellular glutamate level did not increase even with the longer duration of depolarization (**A**). The extracellular glutamate levels in the HT group were significantly lower than those in the NT group (26.2 ± 19.5 vs. 200.1 ± 172.8 μmol/l, *p* = 0.020) (**B**).

The extracellular glutamate levels in the HT group at baseline were 5.6 ± 6.2 μmol/l. In the NT group, peak extracellular glutamate levels elevated in proportion to the duration of depolarization, but in the HT group, peak extracellular glutamate level did not increase even with the longer duration of depolarization (Fig. 6A). The peak extracellular glutamate levels had a significantly lower prevalence than those in the NT group (26.2 ± 19.5 vs. 200.1 ± 172.8 μmol/l, *p* = 0.020) (Fig. 6B).

## Discussion

### Key results

In contrast to the conventional injection model, we developed a model that can control the severity of SAH by controlling ICP. The results of Experiment 1 showed that cortical depolarization occurred in 88.9% of cases, and a duration of depolarization of 20 min or more occurred in 44.4% of cases. The duration of depolarization was significantly correlated with peak extracellular glutamate levels. The extracellular glutamate levels were also significantly correlated with worsening neurological scores and neuronal injury. The results of Experiment 2 showed that cortical depolarization occurred in 100% of cases, but a longer than 20 min duration of depolarization was observed in only 28.6% of cases despite the comparable ICP. CPP recovered earlier in the HT group. The extracellular glutamate levels had a lower prevalence than those in the NT group.

### Early brain injury and physiological parameters

Early brain injury occurs as a result of pathophysiological mechanisms triggered by the combination of mechanical trauma and ischemic injury during the first 72 h after SAH which worsens the outcome. First, when an aneurysm ruptures, the arachnoid space is filled with hematoma and the ICP increases to over 150 mmHg^12^. The compression from the hematoma filling the subarachnoid space causes acute arterial constriction. This, in turn, causes a marked CBF decrease^13^. Subsequently, changes including brain edema, inflammation, oxidative stress, microvascular embolism, CSD, and apoptosis cause EBI^4,14,15^. Our blood injection model using ICP control successfully showed dynamic changes in the membrane potentials, ICP, MAP, CPP, CBF, and extracellular glutamate levels within 1 h after SAH, and these parameters are strongly associated with neuronal injuries.

In a previous study, the duration of depolarization was significantly correlated with the severity of neurological injury^10^. The results of Experiment 1 also indicated that the duration of depolarization has a strong impact on the aggravation of the neuronal injury. In a retrospective study on human SAH, severe ICP elevation was the primary cause of early and prolonged loss of consciousness at the onset of SAH, and loss of consciousness was an important predictor of poor neurological outcome^16^. Loss of consciousness is caused by electroencephalographic suppression due to cortical depolarization, supporting the fact that the longer the duration of depolarization, the worse the neurological outcome.

### Acute vasoconstriction and cerebral autoregulation

Under normal autoregulation, elevation of MAP induces vasoconstriction and reduces the cerebrovascular beds. Next, CBF is maintained in the normal range. In the case of a severely damaged brain in which cerebrovascular autoregulation is disrupted, CBF increases in proportion to the ABP rise by passive dilatation. This leads to brain edema and ICP increase, which results in CPP decrease^17^. In the hyperacute stage of SAH, acute vasoconstriction occurs independently of the changes in ICP and CPP, and is associated with CBF decrease and persistent elevation of extracellular glutamate^18–20^. The subsequent disruption of cerebrovascular autoregulation contributes to EBI^14^. Sukhotinsky et al. modulated CPP by controlling MAP, and examined the influence of CPP on the duration of CSD by KCl application. They showed that induced hypertension significantly shortened the CSD recovery, independent of tissue oxygenation, by facilitating glucose delivery and extracellular K clearance. They concluded that CPP is a critical determinant of CSD duration in the compromised brain, as seen in cases of ischemic stroke and SAH^21^.

The results of Experiment 1 showed that CPP and CBF at 10 min after SAH were significantly correlated with peak extracellular glutamate levels, and extracellular glutamate levels plateaued just 20 min after SAH. This indicates that the impairment of cerebrovascular autoregulation after acute vasoconstriction and the subsequent CBF decrease have a big impact on the prolonged duration of depolarization and neuronal damage. It appears that the fate of SAH patients may be decided within 30 min as a result of ultra-early brain injury.

### Extracellular glutamate levels and therapeutic time window

Under normal conditions, glutamate can be synthesized from glutamine or α-ketoglutarate. After glutamate is released into the synapse, it is removed by excitatory amino acid transporters (EAATs) on the pre- and postsynaptic membranes and also glial cells. EAAT3 is located on the postsynaptic membrane, and EAAT1 and EAAT2 are located on glial cell membranes. Under ischemic conditions, disruptions to Na^+^, K^+^, and pH gradients will cause transporters to function in reverse, leading to elevated extracellular glutamate concentrations^22,23^. The increase in extracellular glutamate levels plays an important role in neuronal injury because the excessive release of extracellular glutamate causes an overload of intracellular Ca^2+^ via post-synaptic N-methyl-D-aspartate (NMDA) receptors. An increase in Ca^2+^ concentration activates Ca^2+^-dependent enzymes, resulting in neuronal injury.

In an experimental severe-ischemic model, extracellular glutamate levels started to rise 4 min after depolarization, and reached maximum levels 12 min after depolarization^24^. In our injection model, we conducted detailed measurements every 2 min. We first showed that the increase in extracellular glutamate levels started 10 min after injection (i.e., approximately 8 min after depolarization), indicating a slower rise in extracellular glutamate levels compared to ischemic injury. Because CBF recovered to 30 to 40% of the baseline after initial mechanical insult and acute vasoconstriction in the SAH model, the elevation of glutamate concentration in the extracellular space might be delayed compared to that in the cerebral ischemic model. In addition, multifactorial mechanisms, such as trapped hematoma, brain swelling, or ICP increase, may also be associated with delayed glutamate release.

Our results indicate that the therapeutic time window in SAH may be longer than that in cerebral ischemia. Fifty percent of neuronal cells were injured around 20 min after injection; therefore, in the experimental model, 20 min might be a therapeutic target that we can aim for to prevent the elevation of extracellular glutamate and neuronal injury.

### Brain hypothermia targeting EBI

The neuroprotective effect of brain hypothermia for SAH has been reported in several studies^25,26^. Recent animal studies have shown that mild hypothermia has the potential to inhibit secondary brain injury by reversing CPP-independent hypoperfusion, preventing brain edema, and reducing vasospasm. Shubert et al. studied the effect of hypothermia using microdialysis in an SAH rat model. They reported that hypothermia ameliorated the acute hypoperfusion and dysfunction of cerebral autoregulation and suppressed the release of excitatory amino acids, such as glutamate, aspartate, and histidine^27^. In another study using a traumatic brain injury model, hypothermia was shown to improve cerebral autoregulation and preserve vascular dilatation in response to hypoperfusion^28^.

Our results of Experiment 2 showed that brain hypothermia within 20 min contributed to the shorter duration of depolarization, earlier recovery of CPP, and reduced elevation of extracellular glutamate concentrations compared to normothermia, despite comparable peak ICP values. Brain hypothermia may inhibit glutamate release in the extracellular space and activate the Na^+^/K^+^/ATP pump by suppressing energy metabolism, inducing early repolarization and preventing brain edema due to the influx of Na^+^ and Ca^2+^. It decreases the activation of downstream signaling for glutamate receptors, especially the overload of intracellular Ca^2+^ that activates Ca^2+^-dependent enzymes, such as protease and phospholipase, leading to the collapse of cytoskeletal elements and arachidonic acid accumulation, respectively. In addition, hypothermia may ameliorate MAP and CPP by suppressing acute vasoconstriction and the subsequent cerebral autoregulation, which may, in turn, facilitate glucose delivery and restore the ionic gradients.

Probit analysis showed that the duration of depolarization that caused 50% of neuronal injury is 22.4 min in a perforation model^10^, and 16.5 min in an injection model. This indicates that the early introduction of neuroprotective therapy such as brain hypothermia within 20 min after SAH inhibits the excessive release of extracellular glutamate and neuronal injuries. Because this study was conducted using rats, the cutoff value of the time window for EBI-targeting therapy in humans remains unknown and further research is needed.

### Limitations

There were several limitations in this study. First, because the observation period was limited to 60 min after SAH, any changes that took place after 60 min were not evaluated. ICP tended to increase during the 60-min period, and it is possible that some cases may have developed brain swelling and herniation after observation, resulting in cerebral ischemia and second depolarization. In addition, because we sacrificed all rats 24 h after SAH in this study, we did not investigate whether the duration of depolarization and elevation of extracellular glutamate levels were associated with chronic neurological deficits and long-term mortality. We also did not evaluate if brain hypothermia could increase the survival rate of the rats. In the future, it is necessary to investigate the effect of brain hypothermia in terms of neuroregeneration, functional recovery, and long-term survival. Second, after 60 min of observation, most cases had brain herniation from the burr hole due to increased ICP, which may have caused cerebral contusion at the bony margin of the burr hole and local glutamate elevation. To minimize the effect of brain herniation, a DC electrode and microdialysis probe were inserted into the same burr hole. Moreover, it is possible that the neuronal damage was caused by the insertion of the electrode or microdialysis probe itself, and we should have established a control group in which only the electrode and microdialysis probe were inserted without SAH. In order to minimize brain damage during probe insertion, we inserted a microdialysis probe gently after incision of pia mater. The pilot study showed that the elevation of extracellular glutamate levels due to probe insertion disappeared within 30 min. Thus, the influence of mechanical injury is not considered to be a concern. Third, in this study, body temperature was managed with rectal and epidural temperatures to avoid brain injury caused by inserting a temperature sensor into the brain cortex, but there was some discrepancy between epidural temperature and brain temperature. In our pilot study, epidural temperature remained under 30 °C during the 1-h observation period, but brain temperature began to increase after pharyngeal cooling was discontinued because of blood flow. Fourth, due to our study design, Experiments 1 and 2 were performed consecutively and not randomized. We selected male rats in this study because female rats have an estrous cycle, and estrogen and progesterone have a neuroprotective effect on ischemic brain injury. Last, in this study, we started brain hypothermia immediately after depolarization, but this is not appropriate for actual clinical situations. To determine the optimum therapeutic time window, we plan to examine when to start hypothermia therapy to prevent glutamate release and neuronal injury.

## Conclusions

We found that a prolonged duration of depolarization correlated with extracellular glutamate levels, and that these two factors worsened the neuronal injury. As the extracellular glutamate levels plateaued within just 20 min after SAH, the therapeutic time window of SAH might be limited. The early induction of brain hypothermia could facilitate the early recovery of CPP, repolarization, and the inhibition of excessive glutamate release, which would prevent ultra-early brain injury following SAH.

## Methods

All experiments were performed in accordance with the National Institutes of Health animal care guidelines and were approved by the Animal Research Control Committee of Okayama University Medical School (approval number: OKU-2019359), and reported in compliance with the ARRIVE (Animal Research: Reporting in Vivo Experiments) guidelines. A flow chart of inclusion and exclusion criteria is shown in Supplementary Fig. 13. In this study, cases in which extracellular glutamate levels could not be measured were excluded. Nine male Sprague–Dawley (SD) rats (Charles River Japan, Yokohama, Japan) weighing 291.7 ± 16.4 g were used in Experiment 1. Seven male SD rats weighing 300.7 ± 27.9 g were used in Experiment 2.

### General procedure

**Figure 7:**
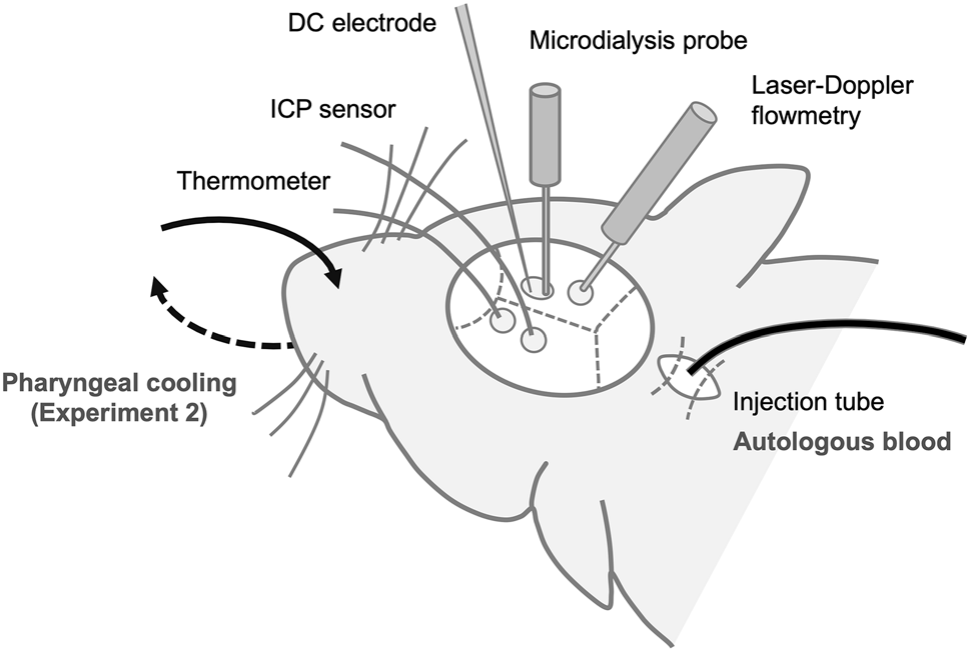
A schematic illustration of the experimental settings.

A schematic illustration of the experimental settings is shown in Fig. 7. Animals were fasted overnight before the experiments, but they had free access to water. Anesthesia was induced with a mixture of 4.0% isoflurane in oxygen. After tracheal intubation and the initiation of artificial ventilation (SN-408-7; Shinano, Tokyo, Japan), anesthesia was maintained with 2.0% isoflurane in 60% oxygen balanced with nitrogen. During the experiment, rectal temperature was maintained at 37.0 ± 0.5 °C with a heated water blanket. A PE50 polyethylene catheter (Sp-45; Natsume Seisakusyo, Tokyo, Japan) was placed in the right femoral artery for continuous monitoring of arterial blood pressure and blood sampling. After placement of the animal in a stereotaxic instrument (Narishige, Tokyo, Japan), four burr holes were drilled into the bilateral parietal lobes. After incision of pia mater, a microdialysis probe (A-I-4-010; Eicom, Kyoto, Japan) was inserted gently through the burr hole in the right parietal lobe (3 mm lateral and 3 mm posterior to the bregma) at a depth of 1250 μm below the cortical surface so that the center of the membrane is located at the fifth layer for the serial measurement of extracellular glutamate concentration. A borosilicate glass direct-current (DC) electrode was inserted at a depth of 750 μm through the same burr hole for monitoring the loss of membrane potentials. To measure regional CBF, a laser-Doppler flow probe (OmegaFlo FLO-CI; Omegawave, Tokyo, Japan) was placed in the ipsilateral burr hole in the parietal lobe. ICP was measured continuously using an ICP sensor (Codman MicroSensor; Codman and Shurtleff Inc., Raynham, MA, USA), which was inserted into the burr hole in the left parietal lobe. Brain surface temperature was also monitored using a small thermocouple (500 μm in diameter) placed in the left parietal epidural space and controlled at 37.0 ± 0.5 °C by a gentle flow of warmed saline coursing over the skull surface.

### Single injection SAH model

A polyethylene catheter connected with a 30-gauge needle (Terumo, Tokyo, Japan) was inserted into the cisterna magna from the occipitoatlantal membrane after the placement of animals in the stereotaxic instrument. Autologous blood was collected and heparinized with 10 units/ml heparin to allow slow injection. Under ICP monitoring, heparinized autologous blood was injected manually so as to reach 80 mmHg in the first 30 s and maintain above 80 mmHg for at least 1 min.

### Brain hypothermia

In Experiment 2, the general procedure and induction of SAH were performed in the same way as in Experiment 1. Brain hypothermia was initiated by the nasopharyngeal cooling method immediately after the onset of loss of membrane potentials. This cooling method is designed to allow heat exchange with the bilateral carotid arteries at the pharynx and enable a rapid decrease of brain temperature without a decrease of body temperature^29,30^. Twenty-gauge cannulas were inserted into the bilateral nasal cavities. Cold physiological saline (4 °C) was infused at a rate of 100 ml/min/kg using a roller pump until the brain surface temperature decreased to 30 °C. Cold saline flowed out of the oral cavity and was aspirated by a suction device. Brain hypothermia was maintained during the 1-h observation period. The animals were rewarmed naturally at room temperature.

### Measurement of extracellular glutamate concentration

The microdialysis probe was perfused with Ringer solution at 2 μL/min using a microsyringe pump (ESP-32; Eicom). Our pilot study showed that the increase in extracellular glutamate concentration caused by probe insertion recovered within 30 min^31^, so measurements of extracellular glutamate concentration were started 40 min after insertion of the probe. The dialysate was automatically collected every 2 min using a fraction collector (EFC-82; Eicom), starting 10 min before blood injection and continuing for 70 min. Quantification of extracellular glutamate concentration in the dialysate was performed using a high-performance liquid chromatography (HPLC) system (Nanospace SI-2; Osaka Soda, Osaka, Japan).

### Neurological and histological evaluation

In Experiments 1 and 2, neurological evaluation was performed 24 h after SAH using an 18-point modified Garcia scale^32^. Next, animals were anesthetized with 4% isoflurane. After insertion of a cannula into the ascending aorta, animals were perfused with heparinized physiologic saline (10 units/ml) and 4% paraformaldehyde. Each DC recording site was marked with blue–black ink through the burr hole. The brain of each animal was removed, embedded in paraffin, and sectioned coronally with a thickness of 5 μm. Sections were stained with hematoxylin and eosin. The number of intact and injured neurons in the fifth layer of the brain cortex was counted at the DC recording site. The pyramidal neurons were characterized by their relatively large somata and their apical dendrites. Pyramidal neurons exhibiting chromatin aggregation in the nucleus, shrinkage, or eosinophilic staining in the cytoplasm were considered to have been injured^24^. We counted normal and damaged neurons in three fields of view with a magnification of 400× and an area of 350 μm × 250 μm. The percentage of neuronal damage was calculated as the number of damaged neurons divided by the total number of neurons in the visual field, multiplied by 100. The evaluation was performed by an observer blinded to this study.

### Statistical analyses

Continuous variables are presented as the mean ± standard deviation. The sample size for correlation analysis was determined assuming α = 0.05, power = 0.80 and a correlation coefficient of 0.7. After assessing the normality using the Shapiro–Wilk W test, the relationship between the physiological parameters (MAP, ICP, CPP and CBF) and the extracellular glutamate levels was analyzed using the Pearson correlation coefficient. Next, probit analyses were performed to identify the duration of depolarization and extracellular glutamate levels that cause 50% of neuronal damage using Origin 8 software (OriginLab Corporation, Northampton, MA, USA). In Experiment 2, these analyses were also performed between Experiment 1 (normothermia [NT] group) and Experiment 2 (hypothermia [HT] group) using Student’s *t-*test (JMP 14 software; SAS Institute, Cary, North Carolina, USA). All tests were two-tailed and *p* values < 0.05 ware considered statistically significant.

## Supplementary Information

Supplementary Information.

## Data Availability

The datasets generated during and/or analysed during the current study are available from the corresponding author on reasonable request.

## References

[1] Hackett ML, Anderson CS (2000). Health outcomes 1 year after subarachnoid hemorrhage: an international population-based study. The Australian cooperative research on subarachnoid hemorrhage study group. Neurology.

[2] Kassell NF, Sasaki T, Colohan AR, Nazar G (1985). Cerebral vasospasm following aneurysmal subarachnoid hemorrhage. Stroke.

[3] Macdonald RL, Weir BK (1991). A review of hemoglobin and the pathogenesis of cerebral vasospasm. Stroke.

[4] Kusaka G, Ishikawa M, Nanda A, Granger DN, Zhang JH (2004). Signaling pathways for early brain injury after subarachnoid hemorrhage. J. Cereb. Blood Flow Metab..

[5] Dreier JP (2009). Cortical spreading ischaemia is a novel process involved in ischaemic damage in patients with aneurysmal subarachnoid haemorrhage. Brain.

[6] Fujii M (2013). Early brain injury, an evolving frontier in subarachnoid hemorrhage research. Transl. Stroke Res..

[7] Suzuki H (2010). Protective effects of recombinant osteopontin on early brain injury after subarachnoid hemorrhage in rats. Crit. Care Med..

[8] Ray B (2019). Systemic response of coated-platelet and peripheral blood inflammatory cell indices after aneurysmal subarachnoid hemorrhage and long-term clinical outcome. J. Crit. Care.

[9] Prunell GF, Mathiesen T, Svendgaard NA (2004). Experimental subarachnoid hemorrhage: cerebral blood flow and brain metabolism during the acute phase in three different models in the rat. Neurosurgery.

[10] Shimizu T (2018). NADH fluorescence imaging and the histological impact of cortical spreading depolarization during the acute phase of subarachnoid hemorrhage in rats. J. Neurosurg..

[11] Karnatovskaia LV, Wartenberg KE, Freeman WD (2014). Therapeutic hypothermia for neuroprotection: history, mechanisms, risks, and clinical applications. Neurohospitalist..

[12] Nornes H, Magnaes B (1972). Intracranial pressure in patients with ruptured saccular aneurysm. J. Neurosurg..

[13] Westermaier T, Jauss A, Eriskat J, Kunze E, Roosen K (2009). Acute vasoconstriction: decrease and recovery of cerebral blood flow after various intensities of experimental subarachnoid hemorrhage in rats. J. Neurosurg..

[14] Conzen C (2019). The acute phase of experimental subarachnoid hemorrhage: intracranial pressure dynamics and their effect on cerebral blood flow and autoregulation. Transl. Stroke Res..

[15] Sehba FA, Pluta RM, Zhang JH (2011). Metamorphosis of subarachnoid hemorrhage research: from delayed vasospasm to early brain injury. Mol. Neurobiol..

[16] Suwatcharangkoon S (2016). Loss of consciousness at onset of subarachnoid hemorrhage as an important marker of early brain injury. JAMA Neurol..

[17] Budohoski KP (2013). Clinical relevance of cerebral autoregulation following subarachnoid haemorrhage. Nat. Rev. Neurol..

[18] Bederson JB (1998). Acute vasoconstriction after subarachnoid hemorrhage. Neurosurgery.

[19] Czosnyka M (1998). Continuous monitoring of cerebrovascular pressure-reactivity in head injury. Acta Neurochir. Suppl..

[20] Westermaier T, Jauss A, Eriskat J, Kunze E, Roosen K (2011). The temporal profile of cerebral blood flow and tissue metabolites indicates sustained metabolic depression after experimental subarachnoid hemorrhage in rats. Neurosurgery.

[21] Sukhotinsky I (2010). Perfusion pressure-dependent recovery of cortical spreading depression is independent of tissue oxygenation over a wide physiologic range. Version 2. J. Cereb. Blood Flow Metab..

[22] Grewer C (2008). Glutamate forward and reverse transport: from molecular mechanism to transporter-mediated release after ischemia. IUBMB Life.

[23] Vandenberg RJ, Ryan RM (2013). Mechanisms of glutamate transport. Physiol. Rev..

[24] Kawase H (2021). Extracellular glutamate concentration increases linearly in proportion to decreases in residual cerebral blood flow after the loss of membrane potential in a rat model of ischemia. J. Neurosurg. Anesthesiol..

[25] Seule MA, Muroi C, Mink S, Yonekawa Y, Keller E (2009). Therapeutic hypothermia in patients with aneurysmal subarachnoid hemorrhage, refractory intracranial hypertension, or cerebral vasospasm. Neurosurgery.

[26] Seule M (2014). Therapeutic hypothermia reduces middle cerebral artery flow velocity in patients with severe aneurysmal subarachnoid hemorrhage. Neurocrit. Care..

[27] Schubert GA, Poli S, Mendelowitsch A, Schilling L, Thomé C (2008). Hypothermia reduces early hypoperfusion and metabolic alterations during the acute phase of massive subarachnoid hemorrhage: a laser-Doppler-flowmetry and microdialysis study in rats. J. Neurotrauma..

[28] Fujita M, Wei EP, Povlishock JT (2012). Effects of hypothermia on cerebral autoregulatory vascular responses in two rodent models of traumatic brain injury. J. Neurotrauma..

[29] Hagioka S, Takeda Y, Takata K, Morita K (2003). Nasopharyngeal cooling selectively and rapidly decreases brain temperature and attenuates neuronal damage, even if initiated at the onset of cardiopulmonary resuscitation in rats. Crit. Care Med..

[30] Takeda Y (2014). Feasibility study of immediate pharyngeal cooling initiation in cardiac arrest patients after arrival at the emergency room. Resuscitation.

[31] Taninishi H (2008). Effect of nitrous oxide on neuronal damage and extracellular glutamate concentration as a function of mild, moderate, or severe ischemia in halothane-anesthetized gerbils. Anesthesiology.

[32] Garcia JH, Wagner S, Liu KF, Hu XJ (1995). Neurological deficit and extent of neuronal necrosis attributable to middle cerebral artery occlusion in rats. Statistical validation. Stroke.

