# Supplementary material for: Depolarization time and extracellular glutamate levels aggravate ultraearly brain injury after subarachnoid hemorrhage
Source: Sci Rep. 2022 Jun 17;12:10256. doi: 10.1038/s41598-022-14360-1 (PMC9205962; doi:10.1038/s41598-022-14360-1)
Supplement: Supplementary file 1 — Supplementary Information. [file 41598_2022_14360_MOESM1_ESM.pdf]

SUPPLEMENTARY MATERIAL

Supplementary table 1. Baseline arterial blood gas in experiment 1

Variables	Experiment 1 (n=9)
Body weight (g)	291.7 ± 16.4
pH	7.43 ± 0.05
PaO2 (mmHg)	227.9 ± 48.7
PaCO2 (mmHg)	37.5 ± 3.3
Hb (g/dl)	13.6 ± 0.7
Glucose (mg/dl)	145.0 ± 35.2

Supplementary table 2. Electrophysiological results after SAH injection in experiment 1

Variables	Experiment 1 (n=9)
Injection volume (ml)	0.34 ± 0.03
Spreading depolarization, n (%)	8 (88.9)
Duration of depolarization (min)	20.6 ± 20.5
duration < 20 min, n (%)	5 (55.6)
duration ≧ 20 min, n (%)	4 (44.4)

Supplementary table 3. Baseline arterial blood gas in experiment 2

Variables	Experiment 2 (n=7)
Body weight (g)	300.7 ± 27.9
pH	7.43 ± 0.03
PaO2 (mmHg)	246.3 ± 47.6
PaCO2 (mmHg)	39.0 ± 2.7
Hb (g/dl)	15.1 ± 0.3
Glucose (mg/dl)	141.6 ± 10.8

Supplementary table 4. Electrophysiological results after SAH injection in experiment 2

Variables	Experiment 2 (n=7)
Injection volume (ml)	0.36 ± 0.04
Spreading depolarization, n (%)	7 (100%)
Duration of depolarization (sec)	13.0 ± 17.8
duration < 20 min, n (%)	5 (71.4%)
duration ≧ 20 min, n (%)	2 (28.6%))

Supplementary figure 1

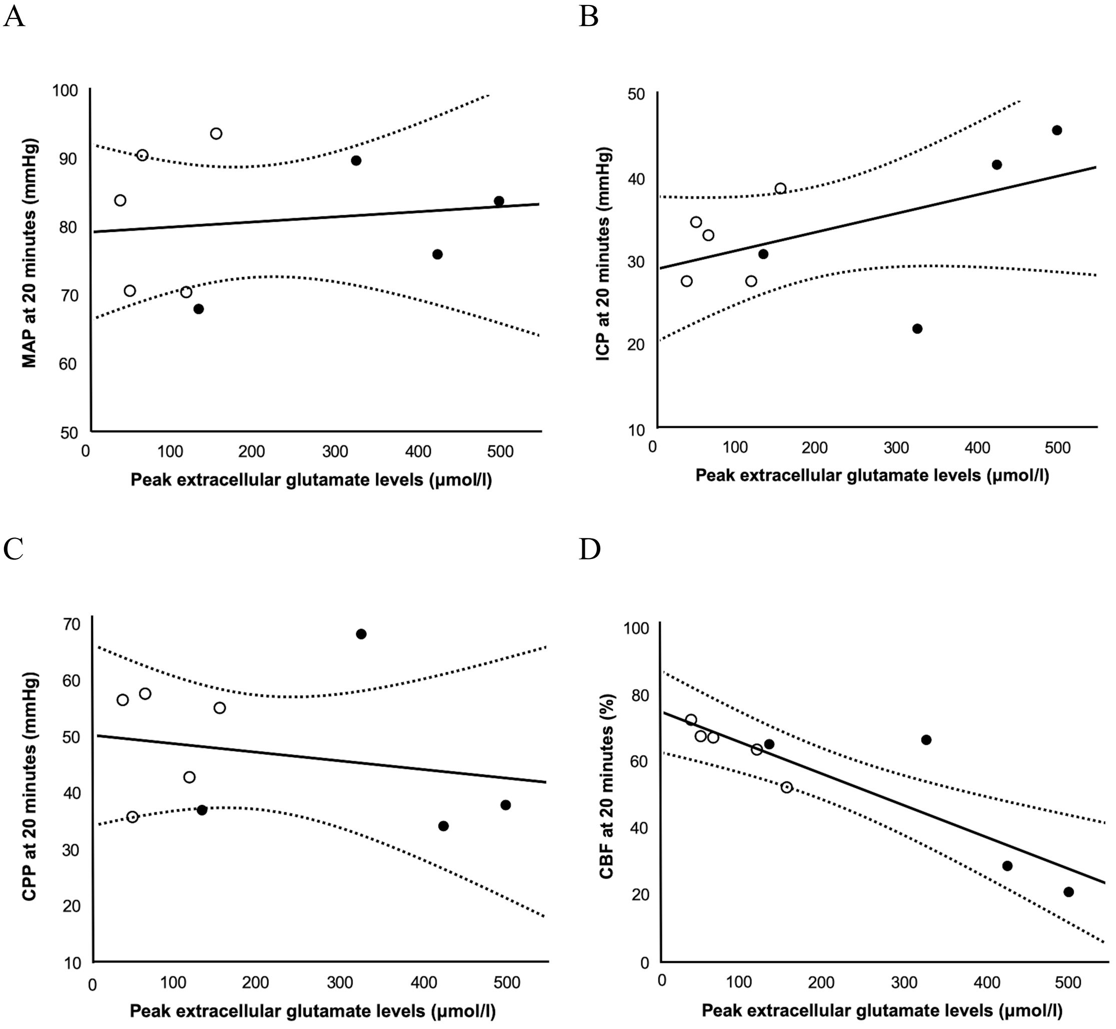

Supplementary figure 2

A

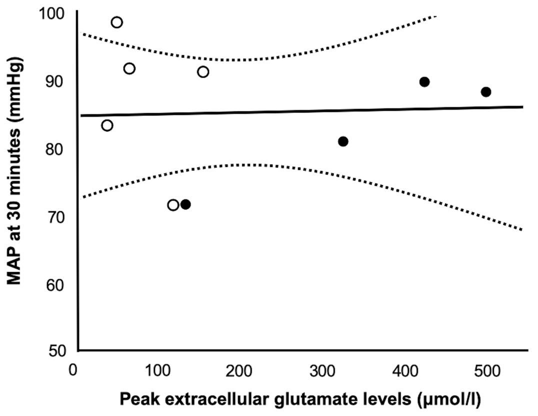

B

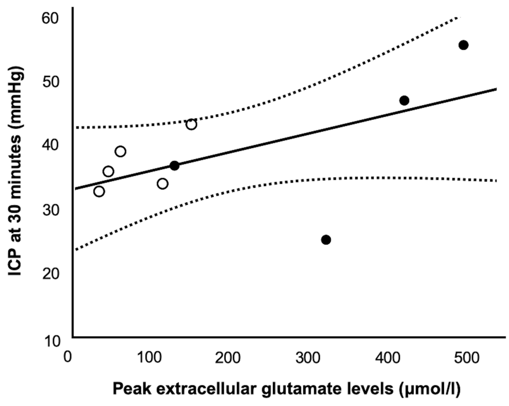

C

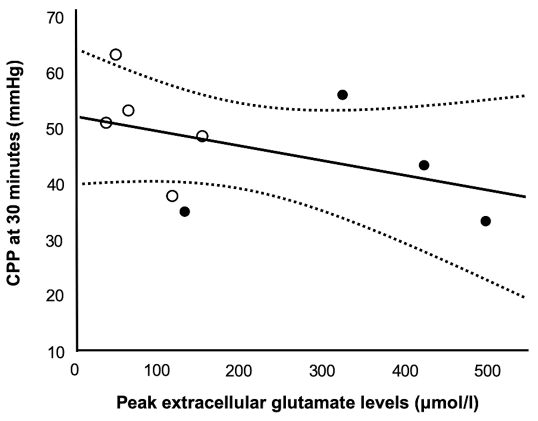

D

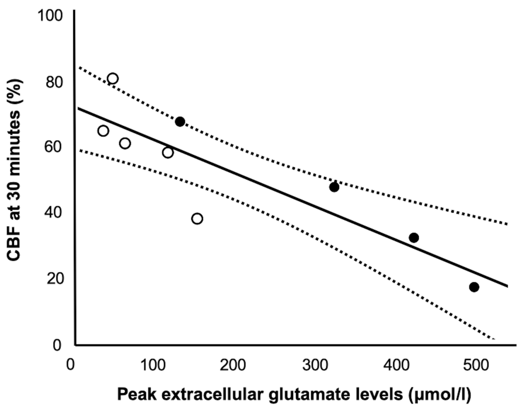

Supplementary figure 3

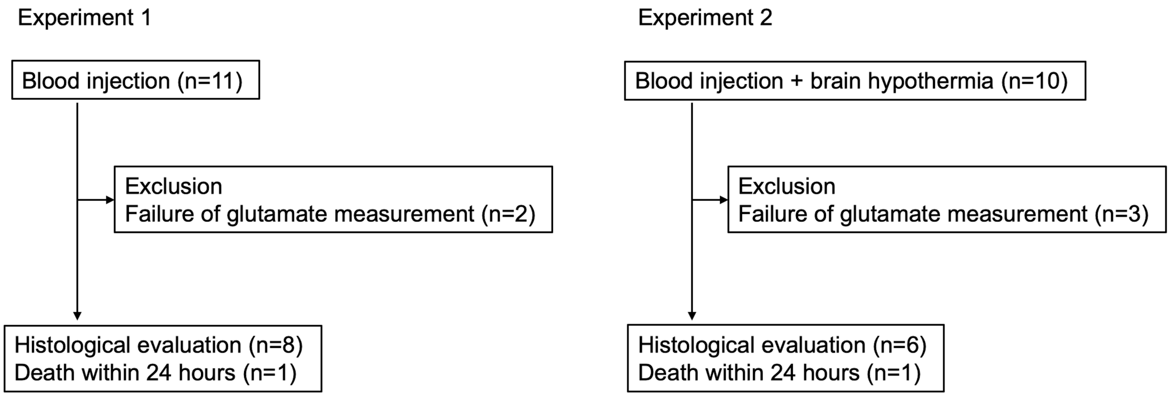

Supplementary Figure legends

Supplementary Figure 1: Linear regression analysis at 20 min showed that only CBF was significantly correlated with peak extracellular glutamate levels (r = -0.87, p = 0.0022) (Black circles indicate a duration of depolarization of 20 min or more, and open circles indicate a duration of depolarization of less than 20 min).

Supplementary Figure 2: Linear regression analysis at 30 min showed that only CBF was significantly correlated with peak extracellular glutamate levels (r = -0.87, p = 0.0020) (Black circles indicate a duration of depolarization of 20 min or more, and open circles indicate a duration of depolarization of less than 20 min).

Supplementary Figure 3: A flow chart of study design. In Experiment 1, 11 rats were subjected to SAH, and 9 rats were included. In Experiment 2, 10 rats were subjected to SAH, and 7 rats were included.
